# Trends in Nanopharmaceutical Patents

**DOI:** 10.3390/ijms14047016

**Published:** 2013-03-27

**Authors:** Adelaide Antunes, Iolanda Fierro, Rafaela Guerrante, Flavia Mendes, Maria Simone de M. Alencar

**Affiliations:** 1 National Institute of Industrial Property (Brazil), Mayrink Veiga, n° 9 Centro, Rio de Janeiro, Brazil; E-Mails: ifierro@inpi.gov.br (I.F.); rafaelaguerrante@gmail.com (R.G.); 2 Federal University of Rio de Janeiro, Postgraduate Program on Technology of Chemical & Biochemical Processes, Av. Horácio Macedo, 2030, Centro de Tecnologia, Bloco I sala I-222, Cidade universitária, Rio de Janeiro, Brazil; E-Mail: flavia@eq.ufrj.br; 3 Oswaldo Cruz Foundation, Institute of Scientific and Technological Communication and Information in Health, Av. Brasil, number 4.365 - Pavilhão Haity Moussatché - Manguinhos, Rio de Janeiro 21.045-360, Brazil; E-Mail: salencar@icict.fiocruz.br

**Keywords:** health, patents, nanotechnology, pharmaceutical

## Abstract

Investment in nanotechnology is now a given constant by governments, research centers and companies in both more developed countries and emerging markets. Due to their characteristics, such as high stability, ability to enable antigen identification on specific cells in the human body and controlling the release of drugs and, therefore, improving therapies, nanoparticles have been the subject of research and patent applications in the pharmaceutical field. According to the Organization for Economic Co-operation and Development (OCDE), patent data can be used as a source of information in order to measure science and technology activities. Thereby, this paper presents an analysis based on patent documents related to nanotechnology in the pharmaceutical sector. As a result, the analysis of patents demonstrate primarily that nanobiotechnology attracts high levels of R&D investments, including nanoparticle-based chemotherapeutic agents/drugs, monoclonal antibody nanoparticle complexes and their role in drug delivery or contrast agents with non-toxic effects.

## 1. Introduction

The global nanotechnology industry was valuing $300 billion (bn) in 2011 and is expected to be worth $1.6 trillion at the end of 2013 and $35,000 bn by 2041. Nanotechnology’s fastest growing sector—nanobiotechnology—is poised for a rapid growth, especially in the field of targeted drug delivery therapies in the oncology sector. The nanobiotechnology market will reach $6 bn by 2017 [1].

There is a myriad of new opportunities in terms of advances in medical science and the treatment of diseases affecting humans. Applications of nanotechnology in medicine and physiology are represented by material and equipment designed to interact with the body on a sub-cellular (or molecular) scale, with a high level of specificity [2]. In more defined terms, nanomedicine in the area of healthcare involves the development of specific new drugs and medicines, in systems of encapsulation and liberation of drugs, diagnostic kits, biosensors, testing devices with images, material for implants, artificial bone and skin, bone substitutes, regeneration of neurons, dental products orthopedic, cardiac and retina implants, to cite just some of the more significant examples already available on the market [3].

It has become increasingly necessary to understand the effects and level of application of biological systems together with nanostructures in the development of research linked with the areas of medicine and health.

The applications of nanobiotechnology allow the creation of material and equipment at the same level as the live organisms, bacteria and even plants. The ability to work on this scale not only provides more control of the biological world, but also leads to empowerment from the use of extraordinary inventions that nature itself has produced after thousands of millions of years of evolution [4].

Not only biomedicines and conventional drugs, such as those used for chemotherapy, but also short strands of interference RNA (gene silencers) are frequently incorporated in vehicles, such as polymers or lipidoid molecules, for the purpose of directing a preferential trajectory in the human organism [5].

The utilization of nanoparticles allows the liberation of a therapeutic agent in a pre-defined location in the organism, the treatment of a huge number of diseases, including diabetes, Alzheimer’s disease, several inflammatory infections, such as arthritis and rheumatism and asthma, as well as cancer.

A plethora of other potential benefits of nanobiotechnology in the area of health can be cited, including:

✓ The new techniques involving molecular images, which allow us to understand the physiological processes of the human body on the nanoscale, thus leading to the development of methods to diagnose and treat diseases more accurately and sensitively. One example in particular is superparamagnetic nanoparticles using proteins for the molecular imaging of cancer;
✓ The new qualitative tools of analysis that will allow for a better understanding of the working of cells at a molecular level and, particularly, the cells related to diseases. The union of proteomic and metabolomic genes into nanobiotechnology will allow for the development of more efficient and specific medicines for each type of disease. Another benefit will be the development of more sensitive and specific *ex vivo* tests;
✓ Nanosensors and nanorobots, which when introduced into the body orally or intravenously, can identify and destroy cancerous cells or those infected by a virus, as well as regenerate damaged issue. These nanorobots can also be used to monitor vital functions (such as measuring arterial pressure or checking the level of glucose in the blood) in patients that require constant medical care.

The use of bibliometric indicators can be an efficient method to analyze research activities. Patent documents are the most important source of technological information, as they contain 71% of all the technology developed around the world, as well as the advantage of being globally standardized. Statistics on patents have been used as indicators of the results of invention-related activities [6]. This paper focuses on patenting as a key indicator of trends in nanobiotechnology.

The aim of this paper is to analyze the main trends of patenting related to nanotechnology applied to the pharmaceutical sector.

### Retrieving Patent Documents Related to Nanotechnology in Pharmaceutical Area

For the recovery of patent documents, the database selected was the Derwent Innovations Index (DII), with the coverage of more than 11 million patent documents filed, published—and eventually granted—from 1963, in more than 40 countries. This database contains patent applications in the areas of chemistry, electricity and electronics and engineering, and its main advantage is the fact that all the patent documents in it are indexed, and their summaries were drawn up by specialists in the area. As such, the summaries are full of keywords and available in English, together with their titles, independent to the country the patent application was filed in and published, which facilitates the recovery of the documents using defined search strategies.

The main reasons for the choice of this patent database were:

✓ The possibility of exporting the patent documents recovered in the search, in their entirety, using software designed to work with this type of information, such as, for example, the commercial software, Vantage Point^®^[7], used in the treatment of the patent data included in this article;
✓ The availability of Manual Codes, a classification attributed in the database to all the patent documents indexed in it. This classification also has a hierarchical structure, such as the International Classification of Patents (IPC) [8], although in less detail. The Manual Codes [9] divide the technological knowledge contained in the patent documents into 21 sections.

For the search of patent documents related to nanotechnology applied to the pharmaceutical sector, the classifications in the Manual Codes were selected as listed below (Table 1), which are specific to the nanotechnology applied to the pharmaceutical sector.

Removing the patent documents that were in more than one of the nine series in Table 1, a total of 7,649 applications were retrieved and imported, using Vantage Point® software. It is important to emphasize that the patent holders can file patent applications in several countries for the same invention. In the case of the present survey, we only considered one patent application filed around the world for each distinct technology (invention).

For the purposes of restricting the series of patent documents obtained, focusing on the special issue “Bioactive Nanoparticle 2012” in the section “Biochemistry, Molecular Biology and Biophysics” of this journal, searches were made in the “abstract” field of the documents, using the keywords listed in Table 2. At this same stage and restricted to the time interval of the series of documents in question, only those that were indexed in the last ten years or between 2002 and 2012 in the Derwent database were selected. The result is shown in Table 2.

The keywords used define different areas of nanobiotechnology, and some are significantly intersected. Of the 17 documents related to the toxicity of nanomaterials, 11 are also contained in the area of nanoparticle toxicity, and the others have a similar focus on this group. In addition, in the areas of nanoparticle cell interaction and nanoparticle protein interactions, the documents of the top-ranked entities filing patent applications are also largely included in the area of nanoparticles used for drug delivery.

Given this, the main focal points and the analysis of the main patent applications filed fall into the following four areas:

✓ Toxicity of nanoparticle;
✓ Nano-imaging agent;
✓ Nanoparticle for drug delivery;
✓ Cancer-targeting nanoparticle.

## 2. Results and Discussion

The analysis was based on the top applicants of patents in the four focal points cited above. Table 3 shows the main entities applying for patents and the number of documents found in each area analyzed in this study. It can be seen that among the 14 main assignees, eight of them are universities and/or research centers and six are companies.

Based on Table 3, one can see that MIT and the University of California are extremely active in the four areas analyzed. It is worth mentioning that one patent document can be included in more than one category. For example, in the case of MIT, of the eight patent applications filed related to the toxicity of nanoparticles, five are also included in nanoparticle used for drug delivery and three in cancer-targeting nanoparticles.

**Figure 1:**
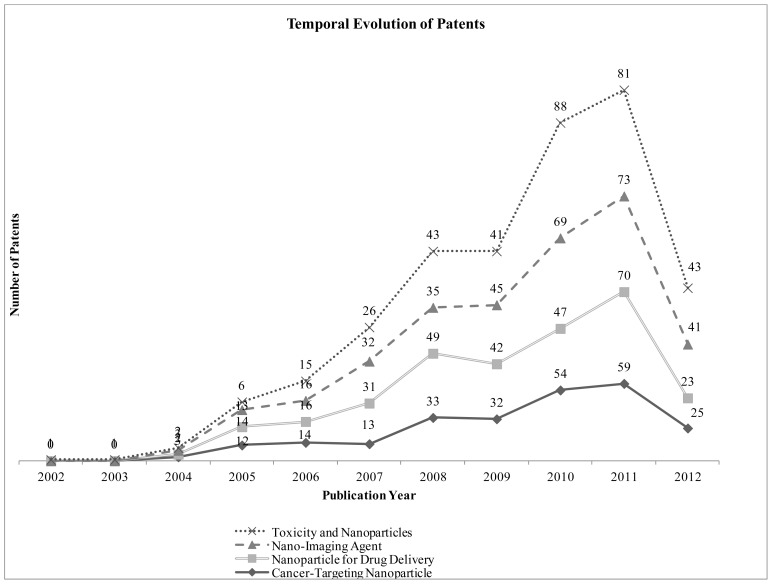
Number of patents per year. Source: Prepared by the authors using data collected from the Derwent Innovations Index. The low number of documents in 2012 is likely explained by the lag in the database consulted, due to the standard period during which the contents of patent applications can be kept secret.

Figure 1 shows a rise in the global interest along the last 10 years in the four main areas analyzed, with a significant growth since 2007 in all areas. It is important to mention the increase of the patents filed related to toxicity and nanoparticles of almost three times between 2006 and 2008 and around double between 2008 and 2010.

The trends of the main applicants for each area in Table 3 are showed below.

### 2.1. Toxicity and Nanoparticles

A total of 347 patent applications were filed under this subject around the world in the last ten years and involved 268 different applicants, either in partnerships or not, of which 216 have single applications. This means only one patent application per applicant.

The leader in patent applications filed in this field is General Electric, with 10 patents, followed by the Massachusetts Institute of Technology (MIT), with eight patents. Four other entities filed seven patents each: the Irish company; Abraxis Bioscience LLC (a Celgene Corporation subsidiary), the French Institute, Centre National de La Recherche Scientifique (CNRS), the Irish Elan Pharma International, Ltd and the University of California, in the United States. Only the patent applications filed by the main institutions were considered in this analysis.

As cited, General Electric (GE) is the company holding the most patent documents under the subject of toxicity and nanoparticles. There are no partnerships in the documents analyzed, and the focus of the use of nanoparticles is related to compositions. The composition for a magnetic resonance imaging contrast agent useful for obtaining a magnetic resonance image (MRI) of a tissue or organ of an animal or human that minimizes toxicity or other discomfort to patients is the main issue in US2006018835 [10]. Along the same lines, the patent applications, US2010278748 and US2012156142, are related to a diagnostic agent composition that comprises nanoparticulate metal oxide, which can form a stable aqueous colloidal suspension that exhibits no substantial change in hydrodynamic diameter, is non-toxic and effectively prevents any agglomeration and oxidation of nanoparticles and exhibits improved biocompatibility, solubility, hydrophilicity and uniformity [11,12]. The patent application, US2010278749, refers to the nanoparticle contrast agent that comprises a core and a shell [13]. The shell comprises silane moieties improving image clearance and is non-toxic. A composition that permits minimal retention of the nanoparticles in the body and decreased toxicity is the focus of patent application, US2010166664 [14]. Furthermore, documents were identified related to methods, as is the case of the applications, US2010278749 and US2010166665, which refer to the method that comprises administering a diagnostic agent composition useful for the diagnostic imaging of a live subject using X-ray, computed tomography (CT) or MRI, where the agent composition contains nanoparticles [13,15]. The nanoparticles provide improved performance and increased blood half-life.

In second place under this topic is MIT, with eight patents, of which two were filed in partnerships. The patent application filed in partnership with Brigham & Women’s Hospital Inc. relates to the new composition comprising a nanoparticle and a biocompatible polymer. The composition is useful in delivering drugs for treating pathologic conditions, e.g., cancer. The composition reduces the amount of a drug present in tissues of the body that are not targeted [16]. The partnership with the University of Northwestern resulted in a patent application related to the composition needed to deliver platinum coordination complex to cytoplasm of, e.g., cancer cells, comprising nanoparticles using a polynucleotide and complex [17]. The patent documents, which were just filed by MIT, are related to, for example: (a) nanoparticles for use in encapsulating a biologically active agent (e.g., polynucleotide, small molecule, bioactive molecule, polypeptide, growth factor or glycosaminoglycan), comprising a quantity of the biologically active agent surrounded by a shell made up of a linear-dendritic hybrid polymer. The advantage is in effectively providing low immunogenicity and toxicity [18]; (b) a drug delivery particle for temporal delivery of two different therapeutic agents. This comprises either a nanocore, including a first therapeutic agent, and an outer layer coating the nanocore containing a second therapeutic agent. This sequential process results in the entrapment of the toxic chemotherapeutic/anti-neoplastic agent within the tumor, leading to increased and selective toxicity against the tumor cells [19]; (c) nanoparticles useful in immune modulation for treating cancer, arthritis, inflammatory diseases, the nanoparticle comprising a polynucleotide and a lipidoid, useful in drug delivery, e.g., enzymes, structural proteins, receptors, soluble receptors, ion channels, cytokines, interleukins, antibodies, antibody fragments, antigens, coagulation factors, albumin, growth factors, hormones, insulin and immunostimulatory RNA [20].

The seven patent applications identified and attributable to Abraxis Bioscience are mainly based on administering compositions comprising nanoparticles (e.g., rapamycin or taxane or paclitaxel), useful for treating pulmonary hypertension, lung cancer, esophagus cancer and that reduce side effects and are non-toxic [21–23].

The Irish company, Elan Pharma, which focuses on the area of biotechnology, has seven patent applications filed under the same topic, with only one in partnership with Alkermes Pharma Ireland Ltd. The patents filed by this company concern, for example, nanoparticulate composition comprising a drug that exhibits chemical stability under environmental conditions, such as prolonged storage periods, and does not require the addition of potentially toxic solvents [24]. Another document, WO2010138539, refers to nanoparticulate injectable composition useful in relieving symptoms and that is non-toxic [25].

Being a research institute, the Centre National de La Recherche Scientifique (CNRS) has partners in six of the seven patent documents selected. Most of these partners are universities or other research centers, such as Université Paris Sud, Lyon University, the University of Versailles Saint Quentin En Yveline, University Fourier Joseph, the University of Strasbourg, the Macromolecular Chemical ASCR Institute, Montpellier University, Rennes University and Institute National Sante & Recherche Medicale. Only one company was identified as a CNRS partner, Ethypharm SA. The patent documents filed by CNRS with its partners, Université Paris Sud and Ethypharm, are related to, for example, biodegradable polymeric nanoparticle composite useful for the manufacturing of a drug used to treat cancer, bacterial or viral systemic infections and the encapsulation and vectorization of molecules having a therapeutic effect. The polymer exhibits less instability and toxicity on non-cancerous cells [26].

The patent documents filed by the University of California in this area did not include any partners and are related to determining the toxic potential of nanomaterials (NMs) and comprises providing a culture of cells, adding a quantity of NMs and performing assays for oxidative stress on the cells. The invention provides test procedures to ensure the safe manufacturing and use of NMs [27]. Another example of the patents filed by the University of California is application, WO2009002386, which is different in that it is presented as identifying biological effects of a nanoparticle on a cell for assessing the cytotoxic effect of a nanomaterial on a cell, measuring the size-dependent biological effect of nanoparticles on a cell and identifying genes [28].

### 2.2. Nano-Imaging Agent

Concerning the patent documents related to the application of nanotechnology in the sector of agents of molecular imagery, 327 patent applications were filed between 2002 and 2012. Of particular note in this universe is the total of 241 patent applications made, either in partnership or not, of which 178 were made by a single entity, and that four entities filed more than five patent applications.

The leader in this field is General Electric, with 20 patents, followed by the University of California, with nine patents, Konink Philips Electronics, with eight, and the University of Texas System, with six.

General Electric (GE) is the company with the most patent documents filed in the field of nanotechnology related to image agents. Of the 20 documents analyzed, only two were made with partners, one with Oxford University (USA) and another with the company, Hammersmith Imanet Ltd. In the patent documents filed by GE alone, the technological focus is mainly related to contrast agents used in magnetic resonance, as in patent application, US2005260137, related to a contrast agent useful for magnetic resonance imaging that comprises several nanoparticles and provides enhanced relaxation, high signal-to-noise ratios and targeting abilities [29]. The contrast possesses resistance to agglomeration, controlled particle size, blood clearance rate and biodistribution. Patent application, US2007025918, is related to a non-toxic contrast agent for enhancing contrast in *in vivo* magnetic resonance imaging measurements and comprises carbon-13-enriched fullerene or carbon nanotube molecules [30]. Patent application, US2007098640, describes a new imaging agent comprised of a passive nanoparticle core and active nanoshell with an active computed tomography contrast agent material, useful in X-ray/computed tomography (CT) imaging [31].

The University of California, second in the ranking in this field, filed nine patent applications, all in partnerships. The abstract supporting patent application, US2006003464, presents a nano-crystal that emits light when energized and an affinity molecule linked to the semiconductor useful for determining the presence of detectable substance in a biological material and for imaging the same sample by both light and electron microscopy [32]. Another example of a patent application filed by this university is WO2009045579, which concerns a probe useful for the detection and treatment of cancer, comprised of a coated nanoparticle attached to an imaging agent used in the manufacture of a medicine for the detection and/or treatment of a cancer, as a nanoparticle-based technology platform for multimodal cancer imaging and therapy that detects cancer by MRI, ESR, PET or near-infrared (NIR) imaging and is capable of detecting cancer cells with greater sensitivity [33]. The multimodal probe is used for *in vivo* imaging and therapy that detects diseased cells by MRI, PET or deep tissue near-infrared (NIR) imaging. Patent application, WO2010096828, is related to a layered nanoparticle comprising a biocompatible layer useful for manufacturing multi-model imaging reagent for imaging living tissue and for treating cancer [34].

Konink Phillips Electronics filed eight patents in the area of nanotechnology associated with image agents, and only one was made in partnership with FEI Corp. As examples, we can cite WO2005070473, which refers to a contrast agent, useful for manufacturing an ultrasound contrast agent comprised of solid metal nanoparticles having an acoustic impedance useful in diagnosing ultrasound contrast imaging and imaging an isolated tissue sample or organ; and patent application, WO2006054240, is related to a contrast agent for medical diagnostic and imaging, comprising metal nanoparticles encapsulated in and/or attached to non-proteinaceous biocompatible or biodegradable matrix particles [35,36].

The University of Texas System filed six patents, with partners in only two, one with the University of Rice William Marsh and the other with the University of Ohio States. Their focus on nanotechnology is mainly linked to imaging agents. For example, patent application, US2008124281, relates to a magnetic resonance imaging contrast agent for molecular imaging applications in cancer [37]. The MRI contrast agent comprises a nanotubular, biocompatible and/or biodegradable carrier. The nanotubular design has precise control of particle size and shape, high superparamagnetic iron oxide payload capacity and prolonged blood circulation time through aligned nanotube orientation with blood flow direction. The abstract of another patent document describes nanoparticles, e.g., a polycation/polyalkyleneglycol glucose conjugate and an active agent that is useful for decreasing the size of tumor in a subject and for the *in vitro* diagnosing of cancer in a subject [38].

### 2.3. Nanoparticle for Drug Delivery

A total of 294 patents were filed in this field by 224 institutions, of which 166 were single entities. Of this last group, approximately 60% were made by universities and/or research centers, suggesting that although still an emerging technology, significant advances have been made over the past few years, with applications in several different areas and that already account for some of the applied research and innovations in the biomedical area. Most of these patents were filed by American institutions, with around 80 of the applications. This data can be corroborated when looking at the timing of the patent filings, excluding those still not published, due to the statutory 12-month grace period for a public disclosure.

The National University of Tsing Hua, in Taiwan, was the leader in terms of patent applications in this field; with 12 filings, of which 11 were made in partnership with GP Medical Inc. Nine of these patent applications involved a composition of nanoparticles, which uses a shell of chitosan, a non-toxic and soft-tissue compatible molecule that enhances diffusion across mucosal epithelia, loaded with the drug to be administered. It is known that hydrophilic medicines, such as peptides and proteins, do not diffuse easily to the blood flow through the intestinal epithelium, and the acid environment in the stomach can degrade these medicines when administered orally, which emphasizes the therapeutic relevance of these new pharmaceutical compositions [39].

In second place was the University of California, with seven patent applications. Patent application, WO2008066965, highlights a manufacturing product that provides biocompatible nanostructures with an increased surface area for enhanced cell and bone growth used in a drug delivery orthopedic device or dental implant, while WO2011143201 presents a new multi-drug conjugate with the main advantage that it controls the molar ratio among multiple drugs and their concentration taken up by the same target cell, optimizing combination therapy regimes [40,41].

The Massachusetts Institute of Technology is next, with a total of six applications, of which two were made in partnership with Brigham & Women’s Hospital Inc. and one with the Children’s Hospital in Boston. Of note among these, WO2008011561, is an application protection for new end-modified poly (beta-amino esters) polymers useful for delivering polynucleotides, contrast agents, RNAi and siRNA for delivering nucleic acids in gene therapy, which have the great advantage that the polymers exhibit improved chemical properties, such as degradation time and ionizability [42]. Another application, WO2008014478, presents a particle useful for the temporal and sequential delivery of two different therapeutic agents with different modes of action or different pharmacokinetics, which can be considered a great therapeutic advantage in the treatment of several chronic diseases [19].

### 2.4. Cancer-Targeting Nanoparticle

A total of 245 patent documents were retrieved in this category. Most of the applications were based on a pharmaceutical composition containing nanoparticles for treatment, not for a specific type of tumor, but several types, including prostate, breast, ovarian, lung, colon, bladder, lymph and melanoma.

The main patent filer is the Massachusetts Institute of Technology, with a total of 11, of which six were made in partnership with Brigham & Women’s Hospital, one with Bind Biosciences Inc., another with Cornell University, one with Northwestern University and, finally, one with the Children’s Hospital in Boston.

In general terms, the patent applications are for new methods and compositions for delivering drugs for cancer or for treating cancer. Patent application, WO2008124639, for example, relates to a controlled-release system comprising target-specific stealth nanoparticles useful for target-specific treatment of a disease and the delivery of a therapeutic agent [43]. It is worth emphasizing patent application, WO2009051837, whose novelty is a nanocarrier for vaccinating subjects with an immunomodulatory agent that is capable of stimulating an immune response in T-cells and in B-cells. Thus, the main use for this composition would be vaccinating a subject that either has or is susceptible to cancer or other diseases [44]. Another original patent application is WO2010101627, which presents a composition containing an agent able to bind a product of a biological cascade and prior to, simultaneously with and/or after the act of administering, activating the biological cascade in the subject. The method is useful to treat benign, pre-malignant and/or malignant tumors and other diseases [45]. A peculiar application, WO2010042555, describes a particle having multiple functionalized surface domains useful for treating disorders, such as cancer, and that is useful in personal care, food products and in agricultural products, including fertilizers, pesticides, fungicides, rodenticides and insecticides [46].

The possible uses cited in the abstracts include not only the treatment of tumors, but also various other diseases. The already cited application, WO2008124639, includes the treatment of atherosclerosis and restenosis, for example [43]. On the other hand, WO2010101627 states that the method claimed is also useful in treating Alzheimer’s disease, Parkinson’s disease, diabetes mellitus, atherosclerosis, asthma and other diseases [45].

The University of California is in second place in this field, with a total of nine patents. Approximately 90% of patent applications are related to the treatment of several types of tumors, using some extremely original ideas. US patent, 2006019900, illustrates the utilization of a protein in cellular adhesion, alpha-4-beta-1 integrin, for treating cancer and an inflammatory or autoimmune disease. The big advantage is that the ligand displays high binding affinity, specificity and stability and is more resistant to cleavage or degradation from proteases found in plasma, gastrointestinal tract and tumor cells [47]. The application, WO2006037081, uses the well-known and already described process of cellular damage, inducing damage to a molecule, which is in or on a cell, comprises delivering nanomaterials to the cells and exposing the nanomaterials to electromagnetic radiation under conditions where the nanomaterials release Auger electrons that cause damage to the molecule [48]. Other patent applications, such as WO2009045579, also involve the detection of tumors, as a multimodal probe used for *in vivo* imaging and therapy that detects diseased cells with greater sensitivity than is possible with existing technologies and targets molecules that localize normal or diseased cells initiating the apoptosis of diseased cells [33].

**Figure 2:**
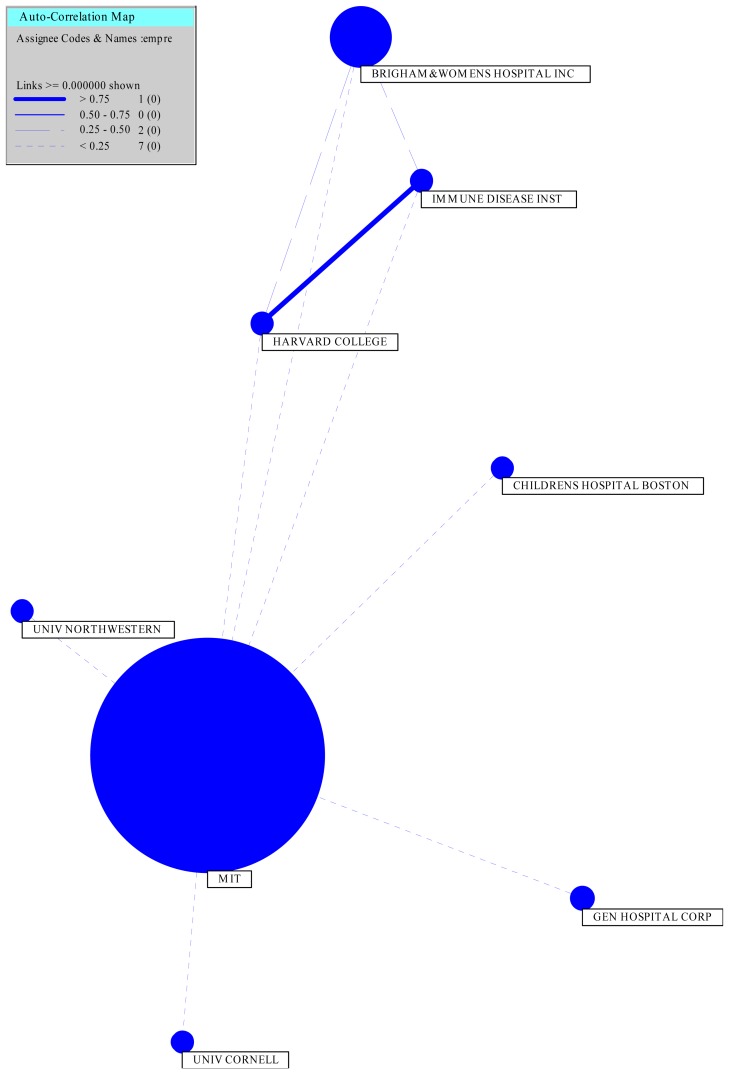
MIT partnerships.

To illustrate the existing partnerships between entities, we drew up the following chart (Figure 2) for the patents applied by MIT and its partners, using the software, Vantage Point ®. It is noteworthy that MIT not only is one of the main applicants in the field, but also the main entity involved in partnerships.

As can be seen in Figure 2, the partners of MIT are the University of Northwestern, the University of Cornell, General Hospital Corporation, Children Hospital Boston, Harvard College, Immune Disease Institute and Brigham & Women’s Hospital Inc. Most partners are universities and/or research centers, showing how research in this area is increasing with the search for innovative products

In the toxicity and nanoparticles area, the partners of MIT are Brigham & Women’s Hospital Inc. and the University of Northwestern. In nano-imaging agents, the patents were filed by MIT with General Hospital Corporation, University of Cornell and Brigham & Women’s Hospital Inc. In nanoparticles for drug delivery, MIT has patents with Brigham & Women’s Hospital Inc. and Children Hospital Boston, and in cancer-targeting nanoparticles, the partners of MIT are the University of Northwestern, the University of Cornell, Children Hospital Boston, Harvard College, the Immune Disease Institute and Brigham & Women’s Hospital Inc. Furthermore, Brigham & Women’s Hospital Inc. has partnerships with the Immune Disease Institute and Harvard College in this area. It is remarkable that the partnership between Harvard College and the Immune Disease Institute is the strongest one, as can be seen by the intensity of the line connecting the two institutions in Figure 2.

## 3. Conclusions

The rising development of nanotechnology in the area of biomedicine can be attributed to the technological advantages, including better distribution profiles of medicines in the organism and directing them to specific organs, which reduces the necessary doses and, consequently, the toxicity and adverse effects. In addition, the possibility of the controlled release of therapeutic agents makes therapy far more efficient and convenient for users, with better patient adhesion to the treatment, particularly those with chronic diseases that require continued administration and repeated doses of medicines.

Also, the toxicity of the nanoparticles and nanomaterial in general is a question that has had an impact in terms of the development of innovations, mainly due to the scarcity of information related to the potential risks to human and environmental health. However, an analysis of the patents that combine toxicity with nanotechnology appears to justify the use of nanoparticles, generally encapsulated as drug delivery or contrast agents, with a non-toxic effect.

One of the main challenges faced in the development and use of medicines for cancer is to ensure that they are concentrated primarily in the affected organs, at selected levels of toxicity in cancerous cells, thus preserving the healthy ones. Due to their reduced size, nanoparticles tend to accumulate in tumors, with less effect on other parts of the organism, which increases the therapeutic efficacy and reduces the main adverse effects caused by chemotherapy, such as vomiting, diarrhea and hair loss.

Dynamic, proactive and socially responsible research will drive nanomedicine as it plays an increasingly integral and transformative role in medicine and public health in the 21st century.

## Figures and Tables

**Table 1:** Manual Codes related to nanotechnology applied to the pharmaceutical sector, and the number of patent documents found.

Derwent manual code	Description	Number of patent documents
B12-M11Q	Nanoparticles	2,306
B12-M10A7	Nanotechnology devices	160
B11-C12	Nanotechnology (general)	5,028
B05-U05A	Nanotubes, nanorods or nanohorns	
B05-U05B	nanofilms	100
B05-U05C	Nanostructures, other than those covered by B05-U05A and B05-U05B	
B05-U05	Other carbon containing 3-D structures	44
B05-U04	Carbon plus heteroatom nanotubes	40
B05-U03	Carbon-only nanotubes	615

Source: prepared by the authors using data collected from the Derwent Innovations Index in May 2012.

**Table 2:** Keywords used in the search, and number of patent documents retrieved.

Keywords	Number of patent documents
toxicity and nanoparticle	341
nano-imaging agent	327
nanoparticle for drug delivery	294
cancer-targeting nanoparticle	245
nanoparticle cell interaction	63
nanoparticle protein interactions	52
toxicity of nanomaterials	17

**Table 3:** The main patent applicant by area.

Total of patents	Entity applying for patents	Toxicity of nanoparticle	Nano-imaging agent	Nanoparticle for drug delivery	Cancer-targeting nanoparticle
23	Univ. of California	7	9	7	9
21	Massachusetts Inst. Technology	8	5	7	11
20	General Electric Co	10	20	0	0
16	Univ. Nat. Tsing Hua	0	0	12	0
15	GP Medical Inc.	0	0	11	0
12	Univ. Texas System	0	6	5	6
8	Univ. Northwestern	0	0	0	6
8	Elan Pharma Int. Ltd.	7	0	0	0
8	Konink Philips Electronics NV	0	8	0	0
7	Centre National de La Recherche Scientifique (CNRS)	7	0	0	5
7	Abraxis Bioscience LLC	7	0	0	0
7	Fuji Film Co Ltd.	0	0	6	0
6	Brigham & Women’s Hospital Inc.	0	0	0	6
5	Univ. Kyushu	0	0	5	0

Univ., University; Inst., Institute; Nat., National. Source: Prepared by the authors using data collected from the Derwent Innovations Index.
